# Heat shock protein 70–2 (HSP70-2) is a novel therapeutic target for colorectal cancer and is associated with tumor growth

**DOI:** 10.1186/s12885-016-2592-7

**Published:** 2016-07-29

**Authors:** Nirmala Jagadish, Deepak Parashar, Namita Gupta, Sumit Agarwal, Vaishali Suri, Rajive Kumar, Vitusha Suri, Trilok Chand Sadasukhi, Anju Gupta, Abdul S. Ansari, Nirmal Kumar Lohiya, Anil Suri

**Affiliations:** 1 Cancer Microarray, Genes and Proteins Laboratory, National Institute of Immunology, Aruna Asaf Ali Marg, New Delhi, 110 067 India; 2 Department of Pathology, All India Institute of Medical Sciences, New Delhi, India; 3 Institute of Rotary Cancer Hospital, All India Institute of Medical Sciences, New Delhi, India; 4 Department of Urology, Mahatma Gandhi Medical College and Hospital, Rajasthan, 302022 India; 5 Department of Pathology, NMC Imaging and Diagnostic Centre, Vidyasagar Institute of Mental Health and Neuro-Sciences, New Delhi, 110065 India; 6 Centre for Advanced Studies, Department of Zoology, University of Rajasthan, Jaipur, 302 004 India

**Keywords:** HSP70-2, Therapeutic target, Gene silencing, Cancer testis antigen

## Abstract

**Background:**

Colorectal cancer (CRC) is the third leading cause of cancer related deaths worldwide both in men and women. Our recent studies have indicated an association of heat shock protein 70–2 (HSP70-2) with bladder urothelial carcinoma. In the present study, we investigated the association of HSP70-2 with various malignant properties of colorectal cancer cells and clinic-pathological features of CRC in clinical specimens.

**Methods:**

HSP70-2 mRNA and protein was investigated expression by RT-PCR, immunohistochemistry, immunofluorescence, flow cytometry and Western blotting in CRC clinical specimens and COLO205 and HCT116 cell lines. Plasmid-based gene silencing approach was employed to study the association of HSP70-2 with various malignant properties of COLO205 and HCT116 cells in in vitro and with tumor progression in in vivo COLO205 human xenograft mice model.

**Results:**

HSP70-2 expression was detected in 78 % of CRC patients irrespective of various stages and grades by RT-PCR and IHC. Our analysis further revealed that HSP70-2 expression was detected in both COLO205 and HCT116 cell lines. Ablation of HSP70-2 expression resulted in reduced cellular growth, colony forming ability, migratory and invasive ability of CRC cells. In addition, ablation of HSP70-2 expression showed significant reduction in tumor growth in COLO205 human xenograft in in vivo mouse model.

**Conclusion:**

Collectively, our results indicate that HSP70-2 is associated with CRC clinical specimens. In addition, down regulation of HSP70-2 expression reduces cellular proliferation and tumor growth indicating that HSP70-2 may be a potential therapeutic target for CRC treatment.

## Background

Colorectal cancer (CRC) is the third leading cause of cancer related deaths in women and second in men in developed countries [1]. Colonoscopy remains the gold standard method for CRC screening till date [2]. CRC patients diagnosed at early stages (stage I & II) have better prognosis with the survival rate of 5 years [3]. However, when diagnosed at later stages (stage III & IV), there are several treatment options but response rates are low and recurrence is high [3]. Chemotherapy is the current therapeutic option for advanced CRC which has limited efficacy with poor prognosis [3]. Therefore, there is a need to identify a tumor associated molecule for developing as a therapeutic target for CRC treatment.

Recently, a member of heat shock protein (HSP) protein family, HSP70-2 has been documented to be associated with bladder carcinoma [4], cervical carcinoma [5], esophageal carcinoma [6] and renal cell carcinoma [7]. HSP70-2 has been proposed to be a new member of cancer testis (CT) antigen family. HSP70-2 has been shown to be expressed in germ cells in testis during spermatogenesis and plays an important role in the first meiotic division of male germ cell and is transcribed from human chromosome 14q24.1, a region also involved in alteration of expression of colorectal cancer-related genes [8]. CT antigens are a unique class of proteins that are expressed in male germ cells and are also expressed abundantly in various malignancies [9].

In the present study, we assessed HSP70-2 gene and protein expression in CRC patient specimens. We demonstrated the involvement of HSP70-2 in various malignant properties of CRC cell line models by employing plasmid driven short hairpin RNA (shRNA) interference approach. Our results showed that HSP70-2 protein is expressed in majority of the early stages of CRC patients. We further documented that HSP70-2 plays an important role in cellular proliferation, migration, and invasion of CRC cells. Also, HSP70-2 shRNA administration reduces the tumor growth of human xenograft in in vivo mouse model. Therefore, HSP70-2 may serve as a potential therapeutic target for better management of CRC patients.

## Methods

### Patient specimens

Colorectal cancer (CRC) patients specimens (*n* = 200) were obtained during surgical tumor resection. The investigations were carried out after obtaining approval of Institute Human Ethical Committee (IHEC approval # 65/11) and of Institutional Ethics Committees of All India Institute of Medical Sciences Hospital (IEC approval # IEC/NP-212/2010) and Mahatma Gandhi Medical College Hospital (IEC approval # IEC/JPR/2012/0170) for providing tissue specimens and clinical data. Duly signed consent forms were obtained from CRC patients enrolled for the investigations. The available adjacent non-cancerous tissue (ANCT) specimens were also collected. Resected tumor specimens were collected in 10 % formalin (fixative) and RNA*later* for IHC and gene expression studies. All tumor specimens were examined by two independent pathologists. Control colon tissue samples (*n* = 40) were obtained from the archives of the Department of Pathology, to investigate the HSP70-2 expression.

### Cell lines

Two colorectal cancer cell lines, COLO205 and HCT116 were procured from American Type Culture Collection (ATCC, Manassas, VA) and were used within four weeks of receiving the cell lines from ATCC. Both cell lines were cultured in recommended medium under standard conditions and were examined for mycoplasma contamination by mycoplasma PCR detection kit (Applied Biological Materials Inc., Richmond, Canada).

### Analysis of *HSP70-2* gene expression in cell lines and CRC specimens

HSPA2 gene expression was examined using RT-PCR as described earlier [4]. Briefly total RNA from CRC tissue specimens and COLO205 and HCT116 cells was isolated using RNeasy mini kit (Qiagen GmbH, Hilden, Germany) according to manufacturer’s guidelines. Synthesis of cDNA was carried out using High Capacity cDNA Reverse Transcriptase kit (Applied biosystems, Foster city, CA). RT-PCR was done using HSPA2 gene specific primers: forward primer- *5’-CCTACTCGGACAACCAGAG-3’*, reverse primer- *5’-TCTCGTCTTCCACCGTCTG-3’.* β*-actin* was used as internal control using β*-actin* specific primers (forward primer- *5’-ATCTGGCACCACACCTTCTACAATGAGCTGCG-3’* and reverse primer- 5’- *CGTCATACTCCTGCTTGCTGATCCACATCTGC-3’*). Further, HSPA2 nucleotide sequence was confirmed by sub-cloning PCR product in TOPO vector (Invitrogen, Life Technologies, Carlsbad).

### Immunohistochemistry (IHC)

HSP70-2 protein validation was performed by employing IHC as described earlier [4]. Paraffin embedded blocks were made from the tissue specimens and serial sections of 4 μm were cut. Briefly, serial sections of CRC tissue specimens and ANCT’s were subjected for HSP70-2 protein localization using anti-HSP70-2 antibody raised in rabbit or control IgG [4]. Subsequently, sections were incubated with horse radish peroxidase (HRP) conjugated goat anti-rabbit IgG (Jackson ImmunoResearch Laboratories, West Grove, PA). The immunoreactivity was visualized using chromogen, 0.05 % 3,3’-diaminobenzidine [(DAB), Sigma Aldrich, St. Louis, MO]. The images of tissue sections were captured using Nikon Eclipse E400 microscope (Nikon, Fukok, Japan) after staining with hematoxylin, and mounted with DPX mountant (Sigma Aldrich, St. Louis, MO).

### HSP70-2 immuno-reactive score (IRS)

Two independent senior pathologists analyzed HSP70-2 immunoreactivity in CRC tissue sections by counting five random fields (>500 cells) under 400x magnification as described earlier [10]. CRC patients tissue specimens were designated as positive for HSP70-2 expression when >10 % of cells expressed HSP70-2 protein.

### Western blotting, Indirect Immunofluorescence (IIF), Fluorescence Activated Cell Sorting (FACS)

Protein expression of HSP70-2 was studied in CRC cells by Western blotting as described earlier [4]. Cell lysate (10 μg) was resolved on 10 % sodium dodecylsulphate-polyacrylamide electrophoresis gel and was transferred onto polyvinylidene fluoride (PVDF) membrane. Immuno-reactivity was carried out by probing the membrane with rabbit anti-HSP70-2 antibody as primary antibody and goat anti-rabbit HRP as secondary antibody and developed using enzyme linked chemiluminescence (Millipore Immobilon Western Chemiluminescent HRP Substrate (ECL), Millipore Corporation, Billerica, USA) reagent. Further, localization of HSP70-2 protein in CRC cells was demonstrated by IIF and FACS as described earlier [11]. Cells were fixed using 3 % paraformaldehyde and permeabilization was done using 0.05 % IGEPAL. Co-localization of HSP70-2 with various sub-cellular organelles was studied by probing the cells with antibodies against organelles [endoplasmic reticulum marker (calnexin, 6D195, sc-70481; Santa Cruz Biotechnology, Santa Cruz, CA), golgi bodies marker (GM130 B-10, sc-55591; Santa Cruz Biotechnology), mitochondria marker (MTCO2, ab3298; Abcam) and nuclear envelope marker (lamin A/C 636, sc-7292; Santa Cruz Biotechnology)]. The images were captured using Carl Zeiss LSM 510 meta confocal microscope (Germany). Co-localization quantification analysis was carried out using AIM4.2 software [12] on LSM Image Browser (Carl Zeiss, Germany). The results are based on Mander’s coefficient of co-localization analyses (M1), calculated from *n* = 20 cells from five examined areas (regions of interest). For FACS analysis, cells were harvested with scrapper and fixed with 0.4 % PFA for 10 min. Fixed cells were then incubated with rabbit anti-HSP70-2 antibody for overnight at 4 °C followed by incubation with secondary antibody. Also, in another experiment, cells were harvested and incubated first with rabbit anti-HSP70-2 antibody for overnight at 4 °C prior to PFA fixation. Unstained cells and cells stained with control IgG were taken as control. Acquisition and analysis was done using Cell QuestPro software on BD FACS CALIBUR (BD Biosciences, California, USA).

### Down regulation of HSP70-2 using plasmid mediated gene silencing

HSP70-2 short hairpin RNA (shRNA) target plasmid (shRNA1, CAT AAC GGT CCC GGC CTA TT; shRNA2, GAG CGG TAC AAA TCG GAA GAT; shRNA3, CGG CGA CAA ATC AGA GAA TGT; shRNA4, TTC GAC GCC AAG AGG CTG CTG ATT) and Control shRNA (NC shRNA, 5’-ATCTCGCTTGGGCGAGAGTAAG-3’) were obtained from Super Array (Sure Silencing shRNA Plasmid, Fredrick, Md). In our initial attempt, we transfected both CRC cell lines (COLO205 and HCT116) with all HSP70-2 shRNA targets and control NC shRNA to examine the efficiency in ablating HSPA2 mRNA by quantitative PCR (qPCR) and HSP70-2 protein by Western blotting as described earlier [4]. The CRC cells were transfected in 6-well plates using lipofectamine reagent (Invitrogen, Life Technologies, Carlsbad, CA) as described earlier [13]. Post 48 h transfection, CRC cells were harvested and cell lysates were prepared for analysis. Two HSP70-2 shRNA targets which resulted in ablation of HSP70-2 protein were used for all subsequent in vitro and in vivo assays.

### Cell growth, viability and colony formation assays

In order to examine the involvement of HSP70-2 expression with cellular proliferation, viability and colony formation ability, both CRC cells were transfected with two sets of HSP70-2 shRNA (shRNA3 and shRNA4) or control NC shRNA. The assays were performed as described earlier [4]. For cellular proliferation, 1x10^4^ HSP70-2 shRNA (shRNA3 and shRNA4) and Control NC shRNA transfected COLO205 and HCT116 cells were seeded per well and counted at 24 h, 48 h and 72 h post transfection. For cell viability assay, 5000 cells were seeded per well in a 96-well plate. After 24 h, 48 h and 72 h of transfection, 3-(4,5-dimethylthiazol-2-yl)-2, 5-diphenyltetrazolium bromide (MTT, Sigma-Aldrich, St. Louis, MO) reagent was added to the media and absorbance was measured at 534 nm after 4 h. For colony formation assay, cells were seeded at different density (400, 800 and 1200 cells). Colonies were allowed to grow and counted after 10 days by staining with toluidine blue.

### Cell migration and invasion assay

Cellular migration and invasion are the key features of cancer cells. Association of HSP70-2 protein expression in cell migration and invasion of CRC cells was performed as described earlier [4]. Briefly, 1x10^5^ cells were counted and seeded onto the 8 μm transwell inserts (BD Biosciences, California, USA) in serum free media for migration assay. For invasion assay inserts were coated with 5 mg/ml matrigel (BD Biosciences, California, USA) and cells were seeded similarly as for migration assay. The cells that migrated or invaded through the insert in the lower chamber were fixed with glutaraldehyde and stained with toluidine blue and counted manually under microscope. The images were captured using Nikon Eclipse E 400 microscope (Nikon, Fukok, Japan).

### Effect of plasmid driven gene silencing in human cancer xenograft model

Total of 20 severely compromised immuno-deficient (SCID) mice [National Institute of Immunology (NII), National Institute of Health] of 6 weeks were kept under sterile NII facility to undertake this study. All investigations in animals were carried out after obtaining ethical clearance from Institute animal ethical committee (IAEC approval #263/11). CRC cells (COLO205) were cultured and counted. Five million cells were injected subcutaneously in the upper portion of hind legs. Animals were monitored regularly and the development of tumor was monitored using caliper, when the tumor volume reached to 50 mm^3^, two groups of mice were made each having 8 mice (Group I: control group and Group II: Experimental group). Three injections, intratumoral schedule of thrice weekly, of 50 μg Control NC shRNA or HSP70-2 shRNA4 were administered into group I or group II respectively. The study was monitored for seven to eight weeks by recording animal weight and measuring tumor volume as described earlier [12]. Subsequently, all animals were sacrificed and tumors were excised for studying HSP70-2 protein and proliferating cell nuclear antigen (PCNA) expression.

### Statistical analysis

Statistical data were analyzed using SPSS20.0 software package (SPSS Inc. Chicago, IL, USA). The statistical difference of HSP70-2 gene and protein expression in different stages, grades, specimens with or without lymph node involvement or metastasis were determined by Mann Whitney *U*-test. Kruskal-Wallis test was performed to find out the significant difference in HSP70-2 amongst various stages and grades. Pearson’s χ^2^-squared test was performed to find out the association of HSP70-2 expression in various stages and grades. A *p* value less than 0.05 was considered statistically significant.

## Results

### *HSP70-2* gene is expressed in CRC cells and specimens

**Fig. 1:**
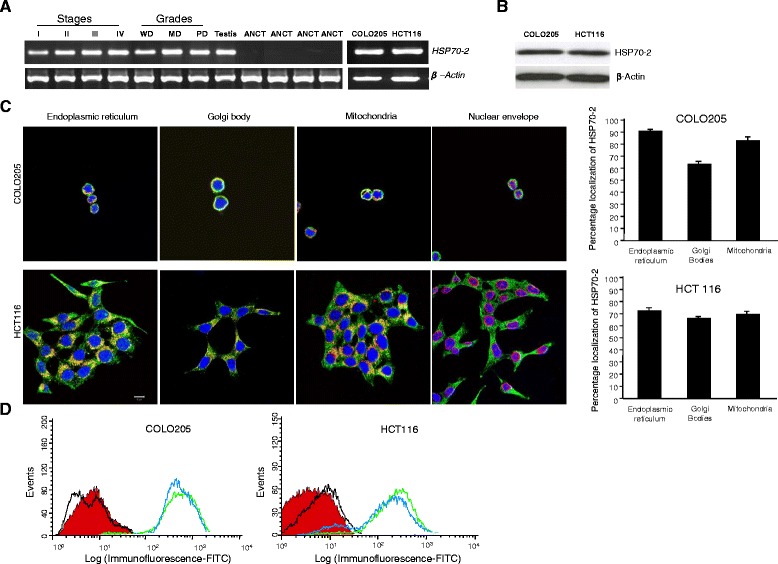
CRC patient specimens and cells express HSP70-2 mRNA and protein. **a** RT-PCR analysis shows *HSP70-2* mRNA expression in stage I-IV, grades WD, MD and PD and CRC cells (COLO205 and HCT 116). ANCT specimens failed to express HSP70-2 mRNA. Testis was used a positive control and β-actin was used as a loading control. **b** Western blotting reveals HSP70-2 protein expression, COLO205 and HCT116 cells. **c** IIF analysis depicts predominantly cytoplasmic localization; co-localization reveals HSP70-2 protein in endoplasmic reticulum, golgi bodies, mitochondria and plasma membrane (yellowish-orange staining). No co-localization was seen with nuclear envelope. Co-localization of HSP70-2 in endoplasmic reticulum, Golgi bodies and mitochondria in both COLO205 and HCT116 CRC cells was observed and quantified (see Methods) Histogram depicts average percentage co-localization in *n* = 20 cells from five examined areas (regions of interest). Original magnification x630, objective x63. Scale bar: 10 μm. **d** Flow cytometric analysis demonstrates the displacement shift of fluorescence (green color peak: depicts cells fixed with PFA prior to first antibody incubation and blue color peak: depicts cells incubated first with primary antibody followed by PFA fixation) in COLO205 and HCT116 cells as compared to control IgG stained (black color peak) and unstained (red color peak) cells. WD: well differentiated, MD: moderately differentiated and PD: poorly differentiated

The HSPA2 gene expression was examined by RT-PCR in CRC tissue specimens and CRC cells (COLO205 and HCT116). RT-PCR data revealed that majority of CRC patients (156 of 200; 78 %) were found positive for HSPA2 gene expression. Both CRC cell lines also expressed *HSP70-2* gene (Fig. 1a). However, no HSPA2 gene expression was detected in ANCT specimens. Among various stages of CRC patient, 75 % of stage I, 78 % of stage II, 79 % of stage III and 76 % of stage IV showed HSPA2 gene expression (Table 1). Further based on histopathological grading, 79 % (56 of 71) of well differentiated and 79 % (81 of 102) of moderately differentiated specimens revealed HSPA2 gene expression as compared to 70 % (19 of 27) of poorly differentiated type. Further, our data revealed that 121 of 155 (78 %) CRC patients with lymph node involvement and 35 of 45 (78 %) CRC patients without lymph node involvement expressed HSPA2 gene. In addition, our data indicated that patients with negative metastatic CRC revealed 117 of 149 (79 %) revealed HSPA2 expression as compared to 39 of 51 (76 %) patients with metastatic CRC.

**Table 1:** Clinicopathological characteristics of colorectal carcinoma patients and HSP70-2 gene and protein expression

Clinicopathological features	RT-PCR (%) of tissue	IHC (%) of tissue	
All tumors	156/200 (78)	156/200 (78)	
Adjacent normal cancerous tissues	0/160 (0)	0/160 (0)	
Tumor stages			
Stage I	6/8 (75)	6/8 (75)	
Stage II	29/37 (79)	29/37 (79)	
Early stages (I + II)	35/45 (78)	35/45 (78)	
Stage III	82/104 (79)	82/104 (79)	
Stage IV	39/51 (76)	39/51 (76)	
Late stages (III + IV)	121/155 (78)	121/155 (78)	
Histologic grades			
Well differentiated	56/71 (79)	56/71 (79)	
Moderately differentiated	81/102 (79)	81/102 (79)	
Poorly differentiated	19/27 (70)	19/27 (70)	
Lymph node involvement			
Positive	121/155 (78)	121/155 (78)	
Negative	35/45 (78)	35/45 (78)	
Metastasis			
Positive	39/51 (76)	39/51 (76)	
Negative	117/149 (79)	117/149 (79)	
Statistical analysis (*p* values of different test used in this study)			
Clinicopathological features	Mann-Whitney *U*-test	Pearson’s χ2 test	Kruskal-Wallis Test
	RT-PCR/IHC	RT-PCR/IHC	RT-PCR/IHC
ANCT, malignancy	0.0001*	0.0001*	-
Tumor Stage			
I + II	0.554	0.835	
II + III	0.152	0.952	
III + IV	0.631	0.737	
I + II & III + IV	0.202	0.967	
I, II, III, and IV	-	0.984	0.573
Histological Grade			
WD, MD+ PD	-	0.587	0.008*
WD + MD	0.388	0.932	
WD + PD	0.09	0.375	
MD + PD	0.03*	0.268	
Lymph Node Positivity	0.202	0.967	
Metastasis Positivity	0.859	0.760	

*IHC* immunohistochemistry, *MD* moderately differentiated, *PD* poorly differentiated, *WD* well differentiated

*p < 0.05, statistically significant

Statistical analysis (*p* values of different test used in this study)

### CRC cells and patient specimens expressed HSP70-2 protein

**Fig. 2:**
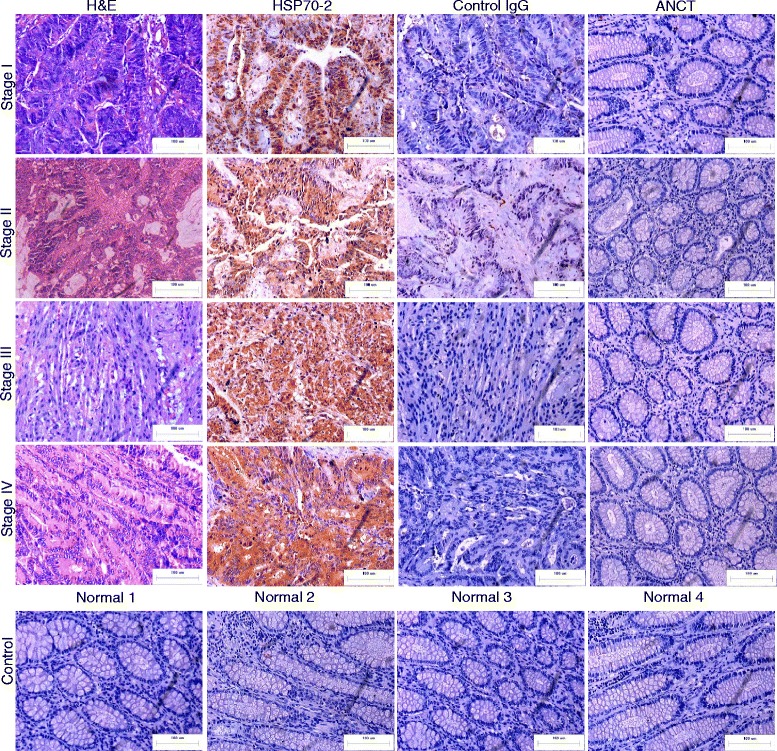
CRC specimens express HSP70-2 protein. First panel shows the cytostructure of representative micrographs of stage I-IV CRC specimen sections stained with H&E. Second panel shows chocolate brown reactivity in Stage I-IV CRC specimen sections probed with anti-HSP70-2 antibody. No immuno-reactivity was observed in stage I-IV CRC specimen sections probed with control IgG antibody (Third panel). ANCT specimens failed to show any immuno-reactivity probed with anti-HSP70-2 antibody (Fourth panel). Bottom most panel shows no HSP70-2 protein expression in representative specimens obtained from healthy patients. Original magnification x200, objective x20. Scale bar: 100 μm

We further validated HSPA2 gene expression for protein expression in CRC cell lines and tissue specimens. Western blotting analysis demonstrated HSP70-2 protein expression in CRC cells (Fig. 1b). HSP70-2 protein expression was examined by indirect immunofluorescence (IIF) in fixed and permeabilized COLO205 and HCT116 cells, which revealed that HSP70-2 protein was detected predominantly in cytoplasm (Fig. 1c). In addition, HSP70-2 protein was also detected in endoplasmic reticulum, golgi body and mitochondria but did not co-localize with nuclear envelope (Fig. 1c). The value of Mander's coefficient show 91.2 % (M1: 0.912), 63.6 % (M1: 0.636) and 83.2 % (M1: 0.832) co-localization of HSP70-2 in endoplasmic reticulum, Golgi bodies and mitochondria respectively in COLO205 cells. Similarly, 73.0 % (M1: 0.730), 66.0 % (M1: 0.660) and 69.4 % (M1: 0.694) co-localization of HSP70-2 in endoplasmic reticulum, Golgi bodies and mitochondria respectively was observed in HCT116 cells. Flow cytometric analyses revealed displacement shift (fluorescence) on X-axis (blue color peak) in COLO205 and HCT116 cells when incubated with anti-HSP70-2 antibody prior to PFA fixation as well as in cells incubated with anti-HSP70-2 antibody post PFA fixation (green peak) as compared to control IgG stained (black color peak) and unstained (red color peak) cells indicating surface localization of HSP70-2 protein (Fig. 1d). Immunohistochemistry (IHC) analyses revealed that majority of CRC patients 156 of 200 (78 %) were found positive for endogenous HSP70-2 protein expression (Fig. 2 and Table 1). However, none of the ANCT specimen demonstrated HSP70-2 protein expression (Fig. 2). CRC specimens probed with control IgG failed to show any immuno-reactivity against HSP70-2. Further, 75 % (6 of 8) of stage I, 78 % (29 of 37) of stage II, 79 % (82 of 104) of stage III and 76 % (39 of 51) of stage IV revealed HSP70-2 protein expression. In different CRC grades 56 of 71 (79 %) well differentiated, 81 of 102 (79 %) moderately differentiated, and 19 of 27 (70 %) poorly differentiated expressed HSP70-2 protein. Our analysis showed that 78 % (121 of 155) of specimens with lymph node involvement and 78 % (35 of 45) of specimens without lymph node involvement showed HSP70-2 protein expression. Our data also indicated that 117 of 149 (79 %) patients with negative metastatic CRC revealed HSP70-2 protein expression as compared to 39 of 51 (76 %) patients with metastatic CRC.

**Fig. 3:**
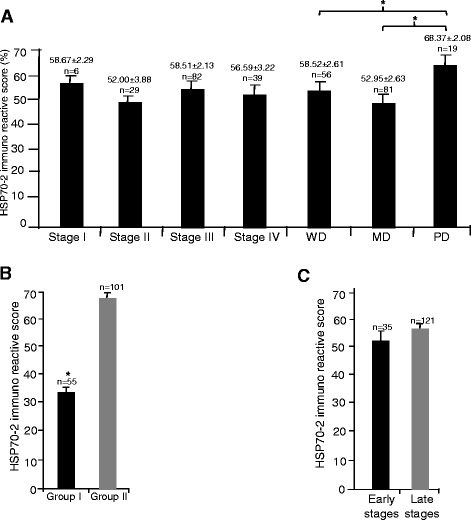
HSP70-2 Immunoreactive Score (IRS). Representative histogram depicts (**a**) HSP70-2 IRS of CRC specimens of stages I-IV and grades WD, MD and PD. **b** HSP70-2 IRS of CRC specimens based on percentage of tumor cells expressing HSP70-2 (group I, <50 % cells expressing HSP70-2; group II, >50 % cells expressing HSP70-2). **c** HSP70-2 IRS of early and late stage CRC specimens. WD: well differentiated, MD: moderately differentiated and PD: poorly differentiated. *p < 0.005, statistically significant

Based on HSP70-2 immuno-reactivity score (IRS), we compared the HSP70-2 IRS among stages and grades. As depicted in Fig. 3, HSP70-2 IRS’s were 58.67 (mean) ± 2.29 (SE) in stage I, 52 ± 3.88 in stage II, 58.51 ± 2.13 in stage III and 56.59 ± 3.22 in stage IV. We also compared early stage (stage I & II) and late stage (stage III & IV) HSP70-2 IRS’s which were 53.14 ± 3.36 and 57.89 ± 1.77 respectively. Kruskal-Wallis test revealed no significant difference among the various stages (*p* = 0.57). Further, in well differentiated, moderately differentiated and poorly differentiated HSP70-2 IRS’s were 58.52 ± 2.61, 52.95 ± 2.63 and 68.37 ± 2.08 respectively. A significant difference was found between moderately differentiated and poorly differentiated (*p* = 0.03) using the Mann Whitney *U*-test, however, no significant difference was observed between well differentiated and moderately differentiated (p = 0.39) or between well differentiated and poorly differentiated (p = 0.09). Interestingly, significant difference was observed among various grades (*p* = 0.008) using the Kruskal-Wallis test. On the basis of lymph node involvement in CRC specimens, HSP70-2 IRS was 57.89 ± 1.77 in patients with lymph node involvement and 53.14 ± 3.36 in patients without lymph node involvement, while with respect to the presence or absence of metastasis, HSP70-2 IRS was 56.59 ± 3.21 in metastatic specimens and 57.11 ± 1.83 in specimens with no metastasis.

We further grouped CRC specimens based on HSP70-2 IRS, group I, low HSP70-2 IRS (<50 % tumor cells expressing HSP70-2) and group II, high HSP70-2 IRS (>50 % tumor cells expressing HSP70-2). Our results indicated that 65 % of CRC patients revealed high HSP70-2 IRS (69.42 ± 1.02) as compared to 35 % with low HSP70-2 IRS (33.97 ± 1.09) which was significantly different was observed among the groups (*p* < 0.0001) as analyzed using Mann Whitney *U*-test, depicted in histograph (Fig. 3).

### Knockdown of HSP70-2 results in reduction of cellular proliferation, cell viability and colony forming ability

**Fig. 4:**
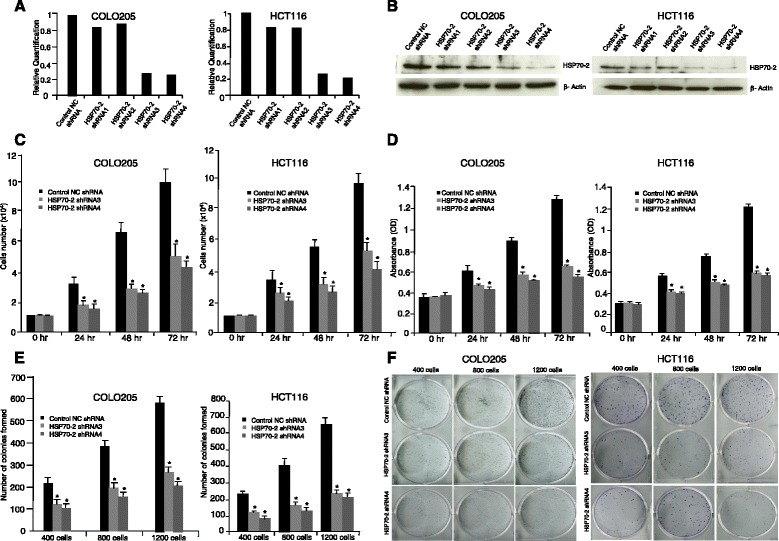
HSP70-2 gene silencing retards cellular growth, cell viability, colony forming ability of CRC cells. **a** Quantitative PCR shows significantly reduced expression of *HSP70-2* mRNA in COLO205 and HCT116 cells when transfected with HSP70-2 shRNA3 and shRNA4 as compared to control NC shRNA or HSP70-2 shRNA1, 2. **b** HSP70-2 shRNA3 & shRNA4 transfected COLO205 and HCT116 cells show significant HSP70-2 protein ablation as compared to control NC shRNA or shRNA1, 2. β-actin was used as a loading control. **c** Cellular proliferation assay demonstrates the significant reduced cellular growth of COLO205 and HCT116 transfected with HSP70-2 shRNA3 and shRNA4 cells at 24 h, 48 h and 72 h. **d** Cell viability as analyzed by MTT assay depicts the significant reduction of viable cells when transfected with HSP70-2 shRNA3 and shRNA4. **e** Colony forming ability was significantly reduced when COLO205 and HCT116 cells were transfected with HSP70-2 shRNA3 and shRNA4. **f** Representative images of the colony forming ability of HSP70-2 shRNA3 and shRNA4 transfected COLO205 and HCT116 cells as compared to control NC transfected cells. *p < 0.0001, statistically significant. All the results are an average of triplicates (*n* = 3) and the experiments were repeated twice

Plasmid driven shRNA mediated gene silencing approach was employed to ablate the HSP70-2 gene and protein by four different HSP70-2 shRNA targets along with control NC shRNA. Quantitative PCR (qPCR) results revealed that HSP70-2 shRNA3 and shRNA4 were most efficient in down-regulating *HSP70-2* gene in COLO205 (72 % and 78 %) and in HCT116 cells (75 % and 80 %) as compared to control NC shRNA (Fig. 4a). Further, Western blotting analysis post 48 h transfection showed maximum ablation of HSP70-2 protein (Fig. 4b) with HSP70-2 shRNA3 and shRNA4. Hence, for all subsequent in vitro assays, HSP70-2 shRNA3 and shRNA4 were used along with control NC shRNA. For cellular proliferation and cell viability assay, CRC cells were transfected with HSP70-2 shRNA3 and shRNA4 which resulted in significant reduction in cellular growth of COLO205 with HSP70-2 shRNA3 (50 %; *p* < 0.01) and with HSP70-2 shRNA4 (56 %; *p* < 0.005) as compared to control NC shRNA transfected cells (Fig. 4c). Similarly significant cellular proliferation reduction was observed in HCT116 with HSP70-2 shRNA3 (44 %; *p* < 0.005) and with HSP70-2 shRNA4 (56 %; *p* < 0.005) (Fig. 4c). In addition, both shRNA targets also revealed significant reduction in cell viability of CRC cell lines as examined by MTT assay (Fig. 4d). Significant reduction in cell viability was observed in COLO205 cells when transfected with HSP70-2 shRNA3 (50 %, *p* < 0.0001) and HSP70-2 shRNA4 (55 %, *p* < 0.0001) as well as in HCT116 cells when transfected with HSP70-2 shRNA3 (51 %, *p* < 0.0001) and HSP70-2 shRNA4 (53 %, *p* < 0.0001) as compared to control NC shRNA. Colony forming ability was also reduced significantly (*p* < 0.05) by 44-55 % (400–1200 cells) for COLO205 cells whereas 44-63 % (400–1200 cells) reduction was observed in HCT116 cells (Fig. 4e).

### Knockdown of HSP70-2 inhibits migration and invasion of CRC cells

**Fig. 5:**
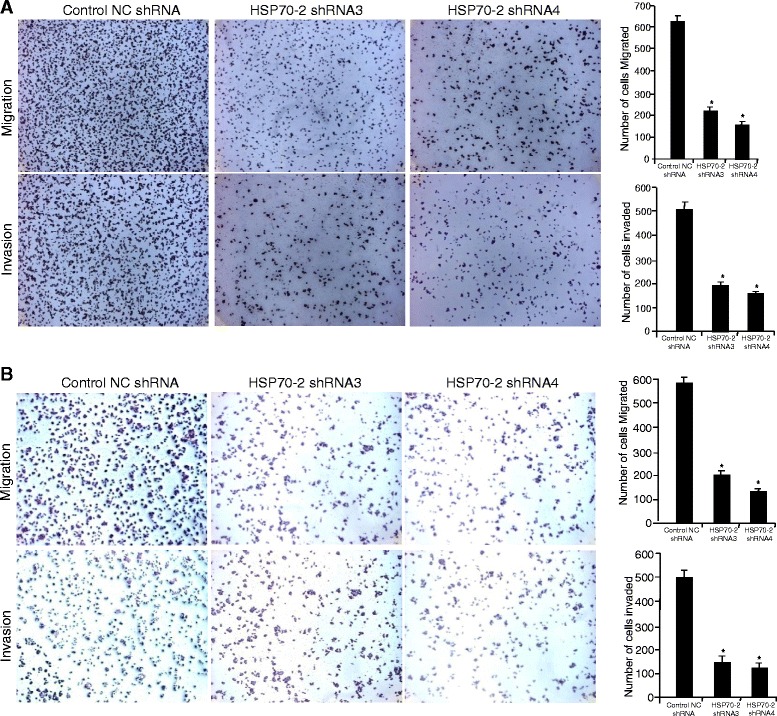
Knockdown of HSP70-2 inhibited migration and invasion of CRC cells. **a** Micrographs depict the reduced migratory and invasive ability of COLO205 post transfection with HSP70-2 shRNA3 and shRNA4 as compare to control NC shRNA. **b** Representative micrograph shows reduction in migration and invasion of HCT116 cells post transfection with HSP70-2 shRNA3 and shRNA4 compare to control NC shRNA. Histogram shows significant reduction in migration and invasion of COLO205 and HCT116 cells. *p < 0.0001, statistically significant, Original magnification x100, objective x10. All the results are an average of triplicates (*n* = 3) and the experiments were repeated twice

Role of HSP70-2 was investigated in migratory and invasive ability that revealed significant inhibition in migration of COLO205 cells when transfected with HSP70-2 shRNA3 (66 %, *p* < 0.0001) and HSP70-2 shRNA4 (74 %, *p* < 0.0001) as compared to control NC shRNA (Fig. 5a). Similarly, significant reduction was observed in HCT116 cells when transfected with HSP70-2 shRNA3 (64 %, *p* < 0.001) and HSP70-2 shRNA4 (74 %, *p* < 0.0005, Fig. 5b) as compared to control NC shRNA. To assess the invasive ability of CRC cells, reconstituted basement membrane (matrigel) was used. Our results revealed a significant reduction of invasive ability (63 %; *p* < 0.005) with HSP70-2 shRNA3 and (67 %; *p* < 0.0001) with HSP70-2 shRNA4 in COLO205 cells (Fig. 5a); (68 %; *p* < 0.001) with HSP70-2 shRNA3 and (70 %; *p* < 0.0005) with HSP70-2 shRNA4 in HCT116 cells as compared to control NC shRNA (Fig. 5b). Our gene silencing studies collectively suggests that HSP70-2 may be involved in migration and invasion of CRC cells.

### HSP70-2 shRNA reduced tumor growth in human colon cancer xenograft in mice

**Fig. 6:**
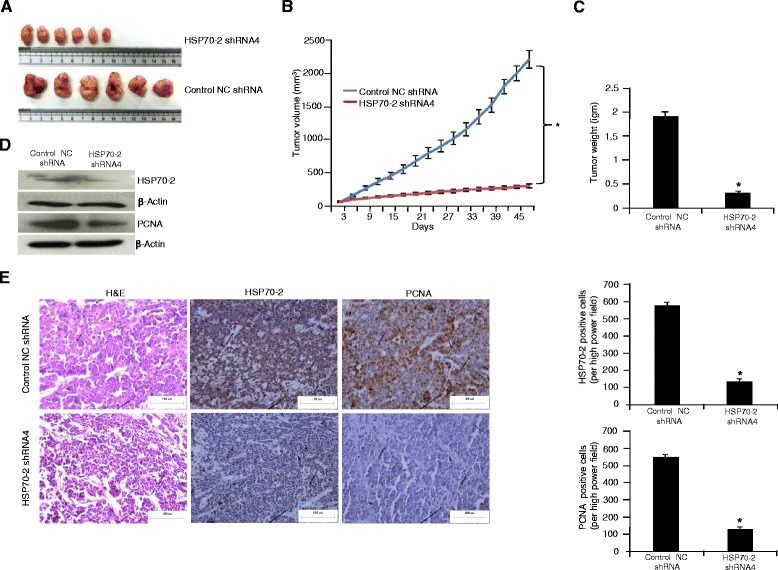
HSP70-2 gene silencing resulted in reduced tumor growth of COLO205 xenograft. **a** Representative images of resected tumor show significant reduction in tumor size treated with HSP70-2 shRNA4 as compared to control NC shRNA treated tumor. **b** Graph represents reduced tumor growth in HSP70-2 shRNA4 treated mice as compared to control NC shRNA treated mice. **c** Histogram depicts decreased tumor weight in HSP70-2 shRNA4 treated mice as compared to control NC shRNA treated mice. **d** Western Blot analysis shows reduced HSP70-2 protein and PCNA expression in HSP70-2 shRNA4 treated mice as compared to control NC shRNA treated mice. β-actin was used as a loading control. **e** IHC analysis of tumor sections show reduced HSP70-2 (middle panel) and PCNA (right panel) expression in HSP70-2 shRNA4 treated mice as compared to control NC shRNA treated mice. Left panel shows cytostructure by H&E staining. Representative histogram shows significant reduced HSP70-2 and PCNA expression in HSP70-2 shRNA4 treated mice as compared to control NC shRNA treated mice. *p < 0.0001, statistically significant. Original magnification x200, objective x20. Scale bar: 100 μm

Our in vitro data indicated that ablation of HSP70-2 protein significantly reduced various malignant properties of COLO205 cells which led us to investigate its effect on COLO205 cell xenograft tumor growth in SCID mice. Control group and experimental group mice were treated with control NC shRNA or HSP70-2 shRNA4 and were observed for 49 days. A representative photograph (Fig. 6a) shows reduced tumor growth in HSP70-2 shRNA4 treated group compared with control NC shRNA treated group. The tumor volume of experimental group mice showed a significant reduction (*p* < 0.0001) in tumor growth as compared to mice administered with control NC shRNA (Fig. 6b). As depicted in histograph, HSP70-2 shRNA4 treatment resulted in 78 % (*p* < 0.0001) decreased tumor growth and 76 % (*p* < 0.0001) tumor weight at day 49 (Fig. 6c). Furthermore, the xenograft tumors were excised and processed for Western blotting and immunohistochemical staining for HSP70-2 protein expression. The HSP70-2 protein expression showed reduction in HSP70-2 shRNA4 treated mice (75 %; *p* < 0.0001) compared with mice treated with control NC shRNA (Fig. 6d and e). Serial tumor sections were also probed for proliferating cell nuclear antigen (PCNA) expression. Our data revealed that there was significant reduction of PCNA expression (75 %; *p* < 0.0001) in tumors treated with HSP70-2 shRNA4 as compared with control NC shRNA shown in Fig. 6d and e. These results suggest that HSP70-2 may be a molecular target for novel cancer treatment.

## Discussion

Colorectal cancer (CRC) is one of the most common causes of cancer related morbidity in both men and women worldwide [14]. It has been well documented that CRC patients diagnosed at early stages have better treatment options with increased survival rate. Therefore, there is a need to identify a tumor marker for early detection and diagnosis. At present, carcinoembryonic antigen (CEA) and carbohydrate antigen (CA19-9) are found to be elevated during late stages CRC when metastasis has already began and are being used in clinical setup with limited efficacy [15]. Cancer testis (CT) antigens are a unique class of proteins that are expressed only in testis during spermatogenesis and have been reported in various malignancies [9]. Only few CT antigens namely sperm associated antigen 9 (SPAG9) [13], OY-TES-1 [16], New York-Esophageal (NY-ESO-1) [17], melanoma-associated antigen 3 (MAGE-A3) [18] and testis specific protease (TSP50) [19] association have been reported in CRC. However, none of these antigens are in clinical practice for CRC detection and diagnosis. Therefore, there is an urgent need to identify a target molecule for early detection and diagnosis and which may also be useful as a therapeutic target for CRC.

HSP70-2, a new member of CT antigens family, is expressed in various malignancies [4, 19, 20]. In the present investigation total of 200 CRC clinical specimens were examined for HSPA2 gene expression which revealed that majority of the patient specimens (78 %) were found positive for *HSP70-2* gene expression irrespective of various stages and grades. It is important to mention that *HSP70-2* gene expression in early stages (I & II) was detected in 78 % of CRC patients. Our laboratory has earlier reported *SPAG9* gene expression in 74 % of CRC patients [13]. OY-TES-1 gene expression was found in 73.3 % of CRC patients [16]. Earlier, MAGE family gene expression was also reported in CRC patients which revealed that 11.6 % of *MAGE-1*, 27.3 % of *MAGE-3* and 22.3 % of *MAGE-4* [21]. The same group also reported SSX family gene expression which showed 5 % of *SSX-1*, 2.5 % of *SSX-2* and 2.5 % of *SSX-4* in CRC patients [21]. Yet another well studied CT antigen *NY-ESO-1* gene expression was only reported in 9.9 % of CRC patients [21]. In contrast our study laid a foundation where HSPA2 gene expression was found in majority of CRC patients which needs to be further validated in large number of specimens.

Recently, differential gene expression profiles have resulted in identification of various genes which are expressed in cancer cells only. However, most of these genes have not been validated for protein expression, hence; have not been put in clinical practice [22]. Therefore, in this context, immunohistochemical analysis was performed to validate *HSP70-2* gene expression and interestingly our data documented that there was no discrepancy found between the HSP70-2 gene and protein expression. HSP70-2 protein was found to be expressed in 78 % of CRC patients. As against gene expression of various CT antigens, protein expression of only few CT antigens has been studied in CRC. SPAG9 protein has been shown to be expressed in 74 % of CRC patients. OY-TES-1 protein was detected only in 43.3 % (26/60) CRC patients [16], whereas, NY-ESO-1 and MAGE-A3 protein is expressed in upto 10 % and 8 % respectively in CRC patients [17, 18]. Yet another testis specific protease (TSP50) was shown to be associated in 68 % of CRC patients. Further, TSP50 expression was not shown to be significantly associated with CRC and the clinicopathological features [19].

CRC disease progression involves tumor growth, migration and invasion of cancer cells to distant sites resulting in metastasis [23]. It was intriguing that HSP70-2 gene and expression was detected in the majority of early stages, late stages and in various grades which prompted us to further investigate the association of HSP70-2 gene and protein in CRC (COLO205 and HCT 116) in in-vitro and in-vivo model. To the best of our knowledge, this is the first study reporting the role of HSP70-2 in CRC specimens and with various malignant properties of CRC cells. Plasmid driven gene silencing approach revealed reduced cellular proliferation, cell viability, colony forming ability, migratory and invasive abilities of COLO205 and HCT 116 cells. In in-vivo human tumor xenograft mouse model HSP70-2 shRNA4 treated animals showed reduced tumor growth. An earlier study demonstrated *HSP70-2* gene expression in primary and metastatic breast cancer in 8 clinical specimens only [20]. We recently demonstrated that HSP70-2 gene silencing inhibited the cellular growth and cell motility in urothelial bladder [4] and cervical cancer [5]. The underlying mechanisms by which HSP70-2 gene and protein silencing alters the malignant properties of CRC are yet not clear. Hence, further studies are warranted to understand the mechanisms through which HSP70-2 is involved in CRC carcinogenesis.

Collectively, our data shows that HSP70-2 is significantly associated with CRC irrespective of its stages and grades. The HSP70-2 gene and protein ablation in CRC cells results in significant reduction of malignant properties. Our study suggests HSP70-2 expression is associated with CRC and may be a putative molecule for early detection and diagnosis and for developing a novel therapeutic target.

## Conclusion

In summary, HSP70-2 plays an important role in various stages and different grades of CRC patients. In addition, HSP70-2 is involved in acquiring various malignant properties of CRC cells and may lead to the development of potential therapeutic target for CRC treatment.

## References

[1] Torre LA, Bray F, Siegel RL, Ferlay J, Lortet-Tieulent J, Jemal A (2015). Global cancer statistics, 2012. CA Cancer J Clin.

[2] Winawer S, Fletcher R, Rex D, Bond J, Burt R, Ferrucci J (2003). Gastrointestinal Consortium Panel. Colorectal cancer screening and surveillance: clinical guidelines and nationale-Update based on new evidence. Gastroenterology.

[3] Scholefield JH, Steele RJ (2002). Guidelines for follow up after resection of colorectal cancer. Gut.

[4] Garg M, Kanojia D, Seth A, Kumar R, Gupta A, Surolia A (2010). Heat-shock protein 70–2 (HSP70-2) expression in bladder urothelial carcinoma is associated with tumour progression and promotes migration and invasion. Eur J Cancer.

[5] Garg M, Kanojia D, Saini S, Suri S, Gupta A, Surolia A (2010). Germ cell-specific heat shock protein 70–2 is expressed in cervical carcinoma and is involved in the growth, migration, and invasion of cervical cells. Cancer.

[6] Zhang H, Chen W, Duan C, Zhang C (2013). Overexpression of HSPA2 is correlated with poor prognosis in esophageal squamous cell carcinoma. World J Surg Oncol.

[7] Singh S, Suri A (2014). Targeting the testis-specific heat-shock protein 70–2 (HSP70-2) reduces cellular growth, migration, and invasion in renal cell carcinoma cells. Tumour Biol.

[8] Son WY, Hwang SH, Han CT, Lee JH, Kim S, Kim YC (1999). Specific expression of heat shock protein HspA2 in human male germ cells. Mol Hum Reprod.

[9] Suri A, Saini S, Sinha A, Agarwal A, Verma A, Parashar D (2012). Cancer testis antigens: A new paradigm for cancer therapy. OncoImmunology.

[10] Agarwal A, Parashar D, Gupta N, Jagadish N, Thakar A, Suri V (2014). Sperm associated antigen 9 (SPAG9) expression and humoral response in benign and malignant salivary gland tumors. OncoImmunology.

[11] Kanojia D, Garg M, Saini S, Agarwal S, Parashar D, Jagadish N (2013). Sperm associated antigen 9 plays an important role in bladder transitional cell carcinoma. PLoS One.

[12] Zinchunk V, Zinchuk O, Okada T (2007). Quantitative co-localization analysis of multicolor confocal immunofluorescence microscopy images: pushing pixels to explore biological phenomena. Acta Histochem Cytochem.

[13] Kanojia D, Garg M, Gupta S, Gupta A, Suri A (2011). Sperm-associated antigen 9 is a novel biomarker for colorectal cancer and is involved in tumor growth and tumorigenicity. Am J Pathol.

[14] Siegel RL, Miller KD, Jemal A (2015). Cancer Statistics, 2015. CA Cancer J Clin.

[15] Kannagi R, Izawa M, Koike T, Miyazaki K, Kimura N (2004). Carbohydrate-mediated cell adhesion in cancer metastasis and angiogenesis. Cancer Sci.

[16] Luo B, Yun X, Fan R, Lin YD, He SJ, Zhang QM (2013). Cancer testis antigen OY-TES-1 expression and serum immunogenicity in colorectal cancer: its relationship to clinicopathological parameters. Int J Clin Exp Pathol.

[17] Jungbluth AA, Chen YT, Stockert E, Busam KJ, Kolb D, Iversen K (2001). Immunohistochemical analysis of NY-ESO-1 antigen expression in normal and malignant human tissues. Int J Cancer.

[18] Jungbluth AA, Busam KJ, Kolb D, Iversen K, Coplan K, Chen YT (2000). Expression of MAGE-antigens in normal tissues and cancer. Int J Cancer.

[19] Zheng L, Xie G, Duan G, Yan X, Li Q (2011). High expression of testes-specific protease 50 is associated with poor prognosis in colorectal carcinoma. PLoS One.

[20] Rohde M, Daugaard M, Jensen MH, Helin K, Jesper N, Jäättelä M (2005). Members of the heat shock protein 70 family promote cancer cell growth by distinct mechanisms. Genes Dev.

[21] Li M, Yuan Y, Han Y, Liu YX, Yan L, Wang Y (2005). Expression profile of cancer-testis genes in 121 human colorectal cancer tissue and adjacent normal tissue. Clin Cancer Res.

[22] Kulasingam V, Diamandis EP (2008). Strategies for discovering novel cancer biomarkers through utilization of emerging technologies. Nat Clin Pract Oncol.

[23] Penna C, Nordlinger B (2002). Colorectal metastasis (liver and lung). Surg Clin North Am.

